# An Analysis of Aesthetic Concerns Identified by Video Conferencing

**DOI:** 10.7759/cureus.31036

**Published:** 2022-11-02

**Authors:** Mansour F Almutairi, Omar M Alhassan, Ahmed I AbdElneam, Adel Alsenaid

**Affiliations:** 1 Department of Dermatology, College of Medicine, Shaqra University, Shaqra, SAU; 2 Department of Internal Medicine, King Saud Hospital, Unaizah, SAU; 3 Department of Biochemistry, College of Medicine, Shaqra University, Shaqra, SAU

**Keywords:** self-perception, covid-19, aesthetic dermatology, zoom, video conferencing

## Abstract

Background

During and after the coronavirus disease 2019 (COVID-19) pandemic, there was a significant increase in the demand for aesthetic procedures nationwide. We believe that one of the factors contributing to this increase was the use of video conferencing apps. Therefore, the purpose of this study was to analyze the aesthetic concerns that were identified by video conferencing.

Methodology

In Saudi Arabia, an observational, cross-sectional study was conducted from February to April, 2022. An online survey was used to assess the aesthetic concerns raised by video conferencing. The survey addressed multiple aspects, including demographic data, information on video call utilization, the attentional focus of the participants during video calls, and new appearance concerns identified on video calls.

Results

The survey successfully captured 432 adult participants. More than 85% of participants disliked at least one character of their appearance during video conferencing. The skin color type (based on the Fitzpatrick scale), time spent on video calls, and visual focus during the virtual meeting were significantly correlated with self-perception of new disliked features.

Conclusion

Video conferencing showed a significant statistical correlation with identified disliked facial/body features in both genders. People with skin color type III were less likely to perceive a disliked feature through video conferencing applications. The likelihood of having a disliked feature was significantly higher among individuals who spent up to one hour per day using video calls. Additionally, those who mainly paid attention to their face or body during virtual meetings were more likely to have disliked features.

## Introduction

Aesthetic and cosmetic procedure demand demonstrates solid global growth. This growth has been observed mainly after lockdown measures were relaxed, together with reinforcement of all necessary post-coronavirus disease 2019 (COVID-19) precautions for a safe environment [1,2]. Different factors attributed to the pandemic influence have been linked to the rise in aesthetic procedures, including the use of video conferencing platforms for work, school, telemedicine, and socialization [1,3].

There was a sudden and dramatic increase in the use of video conferencing applications such as Zoom (Zoom Video Communications, Inc., San Jose, CA), Microsoft Teams (Microsoft, Redmond, WA), and Google Meet (Google, Mountain View, CA), due to the COVID-19 pandemic [4-6]. The rapid rise in the use of video conferencing for communication and utilization of video calls has introduced a virtual hangout in public lives for the foreseeable future.

Besides the popularity and major advantages of video conferencing apps, it has a detrimental effect on how individuals perceive their appearance [7]. Video conferences allow users to have constant access to how they appear, unlike in-person interaction, where people can only take care of their appearance before the meeting. In addition, virtual meetings have shifted the attentional focus toward the facial image instead of being seen from head to toe during an in-person interaction. Furthermore, video calls can run for hours and subsequently generate a mirror effect as people stare at themselves for a long time and are exposed to virtual scrutinizing by themselves and others [8,9]. Mirror gazing is strongly related to selective self-focused attention, which raises self-consciousness and appearance dissatisfaction [10]. Moreover, because of the camera's close proximity, the visual image conveyed during video calls may be unreliable and result in users' distorted perceptions of themselves [11]. Therefore, it was found that users of video calls were highly self-conscious about their facial appearance during the calls [12].

Due to the closure of many cosmetic/beauty services and limited accessibility to aesthetic procedures during the lockdown, people with high dysmorphic concerns were reported to have worsened appearance dissatisfaction [13,14]. Consequently, video conferencing may also have led to the developing of dysmorphic ideas and increased demand for aesthetic and cosmetic procedures. Recent studies looking at how video conferencing affects aesthetic procedures found that new facial concerns were detected due to video conferencing, which was associated with increased interest in aesthetic procedures [15]. In addition, a link between interest in aesthetic procedures and video calls was reported by Chen et al. [16].

Video conferencing will likely be used long after the pandemic because studies indicate that by 2024, only 25% of corporate meetings will be held in person [17]. Consequently, more studies are required to determine the long-term detrimental effects of virtual communication on self-esteem and appearance satisfaction. Accordingly, the association between video conferencing and aesthetic procedure demand in Saudi Arabia is poorly known. Thus, this study aims to analyze the aesthetic concerns identified by video conferencing apps in Saudi Arabia.

## Materials and methods

A cross-sectional study was conducted in Saudi Arabia between February and April 2022. The study received ethical approval from the Medical Research Ethics Committee at Shaqra University, Saudi Arabia (CMD/DWD/SU/2022/03/066). Participants were eligible to participate if they were 18 years or older and currently residing in Saudi Arabia. The sample size was calculated using the SurveyMonkey (Momentive Inc., San Mateo, CA) calculator with a confidence interval of 95% and a margin of error of 5%, which yielded a sample of 385 subjects. Data collection was performed using an online questionnaire to collect the data and was created using Google Forms. The questionnaire was distributed on social media platforms Twitter (Twitter, Inc., San Francisco, CA), Telegram (Tortola, British Virgin Islands), and WhatsApp (Meta Platforms, Menlo Park, CA), with an explanatory cover for participation and written consent.

The questionnaire comprised three main sections. Section A was aimed at gathering data on the demographics of the participants, which included (nationality, age, gender, martial status, employment status, monthly income, and skin color type). Section B aimed to solicit information on video call utilization (average daily time spent on video calls and purpose of video calls). Section C was designed to assess the participants' attentional focus and their new appearance concerns identified on video calls. Participants were asked what they paid most visual attention to during the call, whether or not they had noticed a new aspect of their appearance that they disliked due to video calls, and to specify what these new areas of appearance concern were via checkboxes. Finally, the participants were asked to indicate whether they had engaged in any behaviors related to video manipulation to improve their appearance or to feel more comfortable on video calls.

Statistical analysis

We used SPSS Statistics for Windows, version 25 (IBM Corp., Armonk, NY) for data analysis. We started the data analysis by running frequencies for the demographic information and characteristics of the study sample, followed by running frequencies for the most commonly detected features by the participants as disliked traits to gauge the extent of the problem. Moreover, the adaptive techniques taken by the participants were also analyzed and presented in frequencies. A chi-square test was used to identify the relationship between different variables and their effect on the self-perception of new disliked traits after the increased demands for video conferences. Finally, a logistic regression model was created to measure the direction and the exact effect of statistically significant variables on self-perception.

## Results

The total number of participants in this study was 432, distributed across the different regions of Saudi Arabia. Most of the sample was collected from the central region (50.2%). This study was composed of an almost equal distribution of females and males. Participants' age group mainly fell between 18 and 34 years, representing a total of 74.4%. More than 50% of the participants were employed; more details are given in Table 1.

**Table 1:** Baseline demographics and characteristics of participants who took the survey concerning aesthetic appearance as a result of video calls (n = 432)

Demographics and characteristics	n (%)
Nationality	
Saudi	375 (86.8)
Other Arabs	57 (13.2)
Region	
Central region	217 (50.2)
Western region	132 (30.6)
Eastern region	65 (15.0)
Northern region	6 (1.3)
Southern region	12 (2.7)
Age (years)	
18-24	116 (26.9)
25-34	205 (47.5)
35-44	75 (17.4)
≥45	36 (8.4)
Gender	
Female	233 (55.1)
Male	197 (44.9)
Occupation	
Employed	234 (54.2)
Student	72 (16.7)
Retired/housewife	30 (6.9)
Unemployed	96 (22.2)
Skin color type (Fitzpatrick scale)	
Type I	77 (17.8)
Type II	80 (18.5)
Type III	136 (31.5)
Type IV	93 (21.5)
Type V	28 (6.5)
Type VI	18 (4.2)
Time spent on video calls (duration of average daily time spent on video calls)	
Not on a daily basis	103 (23.8)
<30 minutes	168 (38.9)
30-59 minutes	89 (20.6)
1-2 hours	48 (11.1)
>2 hours	24 (5.6)
Purpose of the video call	
Working/studying	117 (27.1)
Socializing	227 (52.5)
Telemedicine	31 (7.2)
Other reasons	57 (13.2)

This study aimed to analyze whether or not the increased demand for video calls adversely affected participants' perception of their appearance. We found that skin aspects, such as color and pimples, were the most bothering aspects reported by the participants (39.6%). At the same time, nose features, such as shape and size, were the second most disliked aspect identified during the video calls (Table 2).

**Table 2:** Frequency and distribution of new disliked aspects of appearance that participants identified due to the use of video calls (n = 432)

Aspect questioned	Disliked aspects, n (%)	
	No	Yes
Weight/shape (e.g., noticed in chin, cheeks, and/or body)	303 (70.1)	129 (29.9)
Skin (e.g., pimples, color, complexion, and/or wrinkles)	261 (60.4)	171 (39.6)
Nose (shape and/or size)	278 (64.4)	154 (35.6)
Eyes (e.g., shape of eyes, eyelashes, eyebrows, and/or under-eye/dark circles)	331 (76.6)	101 (23.4)
Other (e.g., aging, looking tired, and/or noticing “embarrassing” on-screen behavior)	375 (86.8)	57 (13.2)

Participants had to use some adaptive techniques to improve their appearance or to feel more comfortable during video calls, such as camera position and angle manipulation (45.6%), light adjustment (41.4%), and using in-app filters (39.8%) (Table 3).

**Table 3:** Frequency and distribution of behavior related to video manipulation done by the participants to improve appearance or to feel more comfortable during video calls (n = 432)

Behavior reported	Disliked aspects, n (%)	
	No	Yes
Turning the camera off	366 (84.7)	66 (15.3)
Using a filter on the video cam	260 (60.2)	172 (39.8)
Positioning of self or adjusting the camera angle	235 (54.4)	197 (45.6)
Adjusting the light	253 (58.6)	179 (41.4)

Participants were clustered based on whether or not they disliked at least one aspect of their appearance on video calls (Table 4). We identified that 85% of participants disliked at least one appearance character. Within the 85%, we examined the association between participants' characteristics, such as age and gender, and their newly disliked appearance concerns. We did not find any association of participant's age and gender with the reported new disliked aspects (p = 0.93 and 0.58, respectively). Similarly, marital status, occupation, and monthly income were also not associated with the reported new disliked aspects (p > 0.43).

**Table 4:** Comparison of participants' characteristics between those who at least found one aspect they did not like in their appearance due to video calls and those who did not (n = 432) *The chi-square statistic is significant at 0.05 level. SAR, Saudi Arabian Riyal

			A new aspect of appearance that participants found to be disliked due to the use of video calls		
Characteristic		N	No (n = 63, 14.6%)	Yes (n = 369, 85.4%)	p Value
Age (years)	18-24	116	15 (23.8)	101 (27.4)	0.931
	25-34	205	32 (50.8)	173 (46.9)	
	35-44	75	11 (17.5)	64 (17.3)	
	≥45	36	5 (7.9)	31 (8.4)	
Gender	Female	233	32 (50.8)	201 (54.5)	0.588
	Male	199	31 (49.2)	168 (45.5)	
Marital status	Single	235	34 (54.0)	201 (54.5)	0.941
	Married	197	29 (46.0)	168 (45.5)	
Occupation	Student	72	13 (20.6)	59 (16.0)	0.433
	Unemployed	96	10 (15.9.6)	86 (23.3)	
	Retired/housewife	30	6 (9.5.1)	24 (6.5)	
	Employed	234	34 (54.0)	200 (54.2)	
Monthly income	<5,000 SAR	168	28 (44.4)	140 (37.9)	0.474
	5,000-10,000 SAR	133	19 (30.2)	114 (30.9)	
	11,000-19,000 SAR	88	11 (17.5)	67 (18.2)	
	20,000-30,000 SAR	36	5 (7.9)	31 (8.4)	
	>30,000 SAR	17	0 (0.0)	17 (4.6)	
Skin color type (Fitzpatrick scale)	Type I	77	5 (7.9)	72 (19.5)	0.013^*^
	Type II	80	10 (15.9)	70 (19.0)	
	Type III	136	26 (41.3)	110 (29.8)	
	Type IV	93	20 (31.7)	73 (19.8)	
	Type V	28	2 (3.2)	26 (7.0)	
	Type VI	18	0 (0.0)	18 (4.9)	
Time spent on video calls (duration of average daily time spent on video calls)	Not on a daily basis	103	33 (52.4)	70 (19.0)	0.000^*^
	<30 minutes	168	19 (30.2)	149 (40.4)	
	30-59 minutes	89	4 (6.3)	85 (23.0)	
	1-2 hours	48	6 (9.5)	42 (11.4)	
	>2 hours	24	1 (1.6)	23 (6.2)	
Most of my focus “visually” during the video calls goes to	Both my face and other’s face	177	34 (54.0)	143 (38.8)	0.000^*^
	My own face/body only	83	1 (1.6)	82 (22.2)	
	The other’s face/body only	82	9 (14.3)	73 (19.8)	
	Other on-screen activities	49	3 (4.8)	46 (12.5)	
	Unsure	41	16 (25.4)	25 (6.8)	

On the other hand, the skin color type (based on the Fitzpatrick scale), time spent on video calls, and visual focus during the call were significantly associated with the self-perception of new disliked characteristics (p < 0.01). It is worth mentioning that 52% of the people who reported no new disliked self-aspect had not used video calls on a daily basis.

A logistic regression model was performed to determine the effect of positively correlated factors with self-perceived disliked traits (Table 5). People with skin color type III were less likely to perceive a disliked trait compared to type I by 0.324 (95% CI, 0.10-0.97; p = 0.04). However, people who spent 60 minutes or less were more likely to have an increased chance of having a disliked body feature by 6.242 (95% CI, 1.98-19.67; p = 0.002). Moreover, people who only focused on their face/body during video calls were 15% more likely to have a disliked trait (95% CI, 2.07-119.22; p = 0.008).

**Table 5:** Logistic regression results on the effect of positively correlated values on a new aspect of appearance that participants found to be disliked due to the use of video calls *Statistically significant. ^a^The outcome for this variable was not possible to calculate by the model.

Variable		N	Odds ratio (CI)	p value
Skin color type (Fitzpatrick scale)	Type I	77	Reference	
	Type II	285	0.463 (0.135–1.588)	0.221
	Type III	136	0.324 (0.108–0.973)	0.045^*^
	Type IV	27	0.336 (0.109–1.035)	0.057
	Type V	6	1.021 (0.169–6.171)	0.982
	Type VI^a^		—	—
Time spent on video calls (duration of average daily time spent on video calls)	Not on a daily basis	103	Reference	
	<30 minutes	168	3.262 (1.63–6.527)	0.001^*^
	30-59 minutes	89	6.242 (1.98–19.677)	0.002^*^
	1-2 hours	48	2.412 (0.866–6.721)	0.092
	>2 hours	24	6.79 (0.818–56.345)	0.076
Most of my focus “visually” during the video calls goes to	Both my face and other’s face	177	Reference	
	My own face/body only	83	15.737 (2.077–119.229)	0.008
	The other’s face/body only	82	1.377 (0.59–3.212)	0.459
	Other on-screen activities	49	2.377 (0.656–8.615)	0.188
	Unsure	41	0.583 (0.258–1.316)	0.194

## Discussion

The demand for aesthetic procedures has increased more than usual since the COVID-19 crisis. Simultaneously, there was a dramatic increase in using video conferencing applications, such as Zoom and Microsoft Teams. In Saudi Arabia, government and private services, such as education, work, and telemedicine, were switched from in-person interaction to virtual platforms using the applications mentioned earlier. However, the association between the increased demand for aesthetic procedures and virtual conferencing has not been investigated in Saudi Arabia. In this study, we aimed to analyze the aesthetic concerns identified by using virtual meeting applications. We believe that the aesthetic concerns identified through video conferencing are one of the factors beyond the surge of aesthetic procedures. Several studies have proven that staring at self-image is linked with appearance dissatisfaction [18-20]. Thus, video conferencing may increase the chance of staring at self-image and subsequently noticing more disliked facial/body features.

In our study, we found that 85.4% of the participants identified at least one disliked feature through video conferencing, and the vast majority turned the camera on during the meetings. Thus, they were exposed to self-image staring and identifying new disliked facial/body aspects. Furthermore, we found that visual focus during virtual meetings was a significant factor associated with identifying a disliked feature. The participants who were focusing only on their faces were 15% more likely to have aesthetic concerns relative to those who were focusing on others or screen activities.

In the current study, most of the disliked features identified by video conferencing were associated with facial appearances, such as skin blemishes (i.e., pimples and color) and the nose (i.e., shape and size). That is not surprising as the above-shoulder area is the most visible area during video conferencing calls. A recent study found that public interest in aesthetic procedures for the above-shoulder area has significantly increased after COVID-19 in the United States [21]. This finding validates that staring at self-image correlates with appearance dissatisfaction and supports the hypothesis that video call users are more prone to having aesthetic concerns related to their facial features.

Our study revealed that participants’ age was not correlated with a new disliked feature due to using virtual meeting applications. This suggests that the identified disliked features were not related to aging changes and were probably due to other cosmetic reasons.

It was recognized that females tended to be more interested in cosmetic procedures and to have aesthetic concerns about their appearance based on studies done in Saudi Arabia before the COVID-19 pandemic [22-24]. However, our survey did not identify a significant association between gender and aesthetic concerns. Among female participants 86%, and among male participants, 84% reported at least one disliked facial/body feature. Similarly, few recent studies published during and after the COVID-19 pandemic in Saudi Arabia found no significant association between gender and cosmetic procedure demand [25,26]. Thus, a noticeable aesthetic concern was found among male participants, which could be explained by the video conferencing impact on appearance satisfaction. As a result, virtual meetings significantly increased appearance concerns among both genders.

Our data revealed that the average daily time spent on virtual meetings was a significant factor associated with identifying a disliked facial/body feature. We found that people who spent less than 30 minutes a day were 3% more likely to have a disliked facial/body feature relative to participants who were not using virtual applications on a daily basis. The risk increased to 6% in those who spent up to one hour daily. The risk was more with people who spent more than two hours, but not significant relative to non-daily users. However, there is a variation between studies regarding the duration of time using virtual applications and appearance dissatisfaction [8,9,15,27].

Skin color type (based on Fitzpatrick scale) was the third correlated factor with self-perceived disliked features. Interestingly, participants with skin color type III were 32% less likely to perceive a disliked feature compared with type I. In contrast, participants with skin color type V were more likely to have a disliked feature, but it was not statistically significant. The reason behind that was not understood, which may need further investigations in terms of aesthetic concerns and dysmorphic disorders.

This study also had some limitations. Body dysmorphia was not addressed through our survey; hence, those who were focusing on themselves during virtual meetings could be comorbid with body dysmorphia. Further investigation on the effect of video conferencing on those already diagnosed with body dysmorphia is necessary. Another limitation we can address here is the unreliable results associated with the Fitzpatrick skin type and Saudi population due to the self-assessment nature used in the online questionnaire.

## Conclusions

During the COVID-19 pandemic, video conferencing usage significantly increased; the demand for aesthetic procedures was found to be increased as well. Our study aimed to analyze the aesthetic concerns identified through video conferencing applications. We found that video conferencing correlated significantly with identified disliked traits, as 85.4% of participants identified at least one disliked facial/body feature regardless of age and gender. Moreover, skin color type (based on the Fitzpatrick scale), time spent on video conferencing, and visual focus during the call were significantly associated with self-perception of a new disliked feature. This study encourages the scientific society to conduct more research on this topic, primarily focusing on possible comorbid body dysmorphia.
